# Outcomes of 50 Patients With Ewing Sarcoma Family of Tumors Treated at a Single Institution in Taiwan

**DOI:** 10.1097/MD.0000000000003830

**Published:** 2016-06-03

**Authors:** Chih-Ying Lee, Chueh-Chuan Yen, Hsiu-Ju Yen, Cheng-Ying Shiau, Ta-Chung Chao, Po-Kuei Wu, Cheng-Fong Chen, Paul Chih-Hsueh Chen, Hung-Ta Hondar Wu, Hong-Jen Chiou, Chao-Chun Chen, Giun-Yi Hung, Wei-Ming Chen

**Affiliations:** From the Division of Pediatric Hematology and Oncology (C-YL, H-JY, C-CC, G-YH), Department of Pediatrics, Taipei Veterans General Hospital; School of Medicine (C-YL, C-CY, H-JY, C-YS, T-CC, P-KW, C-FC, PC-HC, H-THW, H-JC, C-CC, G-YH, W-MC), National Yang-Ming University; Therapeutical and Research Center of Musculoskeletal Tumor (C-YL, C-CY, H-JY, C-YS, T-CC, P-KW, C-FC, PC-HC, H-THW, H-JC, G-YH, W-MC), Department of Orthopedics; Division of Medical Oncology (C-CY, T-CC), Department of Oncology, Taipei Veterans General Hospital; Department of Life Science (H-JY, G-YH), National Taiwan Normal University; Division of Radiation Oncology (C-YS), Department of Oncology; Department of Orthopedics (P-KW, C-FC); Department of Pathology (PC-HC); Department of Radiology (H-THW, H-JC), Taipei Veterans General Hospital; National Defense Medical Center (H-JC), Taipei; Department of Pediatrics (C-CC), Taipei Veterans General Hospital Hsinchu Branch, Hsinchu; and Rehabilitation and Technical Aid Center (W-MC), Taipei Veterans General Hospital, Taipei, Taiwan.

## Abstract

To identify the prognostic factors and long-term outcome of the Ewing sarcoma family of tumors (ESFT), data on 50 patients with ESFT treated at Taipei Veterans General Hospital between February 1991 and March 2014 were retrospectively considered. The influence of patient demographics, tumor features, and clinical and therapeutic parameters on overall survival (OS) and progression-free survival (PFS) rates were assessed. The results revealed that 21 of the 50 patients (42%) were metastatic at diagnosis. The median follow-up time was 1.8 years. The 5-year OS and PFS for patients who were nonmetastatic were 61.6% and 55.5%, respectively, and 18.8% and 15.4% for patients who were metastatic, respectively. The key adverse prognostic factor was metastasis at diagnosis. Radiotherapy for local control was associated with improved PFS. The high rate of primary metastasis and poorer outcomes of nonmetastatic ESFT compared with results from Western studies, along with previously reported low rates of ESFT in Taiwanese people, suggest that genetic factors play a role in the pathogenesis of ESFT and chemotherapy pharmacokinetics and pharmacodynamics. Radiotherapy in local treatment should be considered more aggressively in Taiwanese patients with ESFT.

## INTRODUCTION

The Ewing sarcoma (ES) family of tumors (ESFT) are malignant small round blue cell tumors that include ES of bone, peripheral primitive neuroectodermal tumor (pPNET), and extraosseous Ewing tumor (EES). These cancers can develop in the bones or nearby soft tissue, and tend to have the same cytogenetic translocation, t (11;22)(q24;q12). They are grouped for both treatment and prognostic factor analysis.

ES is regarded as a malignancy in children and adolescents. Although rare, it is the second most common type of bone cancer among people aged <20 years.^1,2^ Strong racial disparities in the incidence rates of ES have been reported previously.^3–6^ Taiwan has a low rate of ES compared with the rate in the United States and England;^5^ in a similar manner, low rates have also been reported in China and Japan.^6^ Because of the rarity of the disease, studies on the treatment outcome and prognostic factors of ES in East Asia are more difficult to conduct. In Taiwan, only 1 previous study was performed, reporting the outcome of 12 patients with ES with a 2-year survival rate of 45.5%.^7^ By extending the study period over 23 years, we aim to identify the prognostic factors and evaluate the long-term outcome of ESFT at a single institution, to provide an overview of this rare cancer in Taiwanese people, and to help develop risk-adapted therapeutic strategies for treatment in the future.

## METHODS

### Patients

From February 1991 to March 2014, patients who were diagnosed with ESFT (i.e., ES of bone, pPNET, and EES) and treated at Taipei Veterans General Hospital (TVGH) were retrospectively evaluated. Patient characteristics and cancer-related variables are listed in Table 1.

**TABLE 1:**
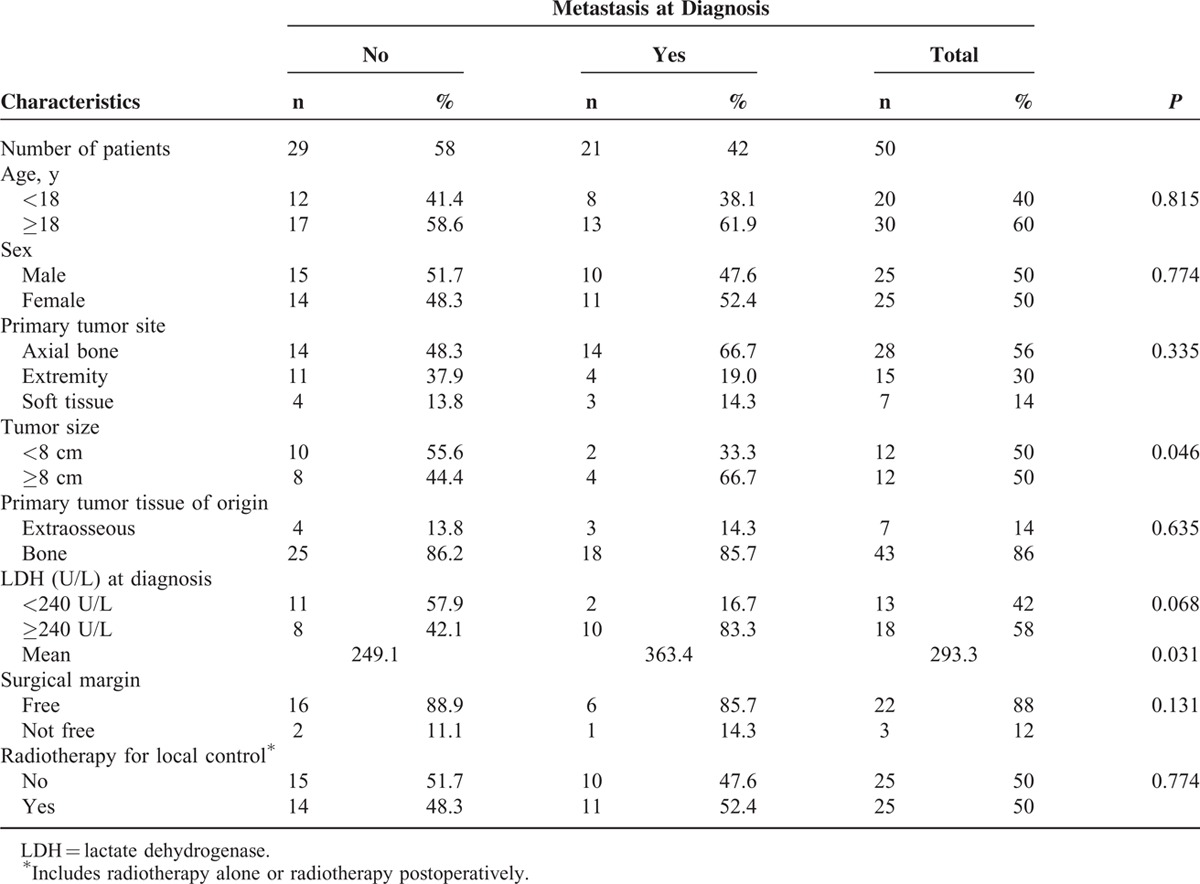
Patient Characteristics and Tumor Features of ESFT (n = 50)

### Clinical Staging and Follow-Up

A complete medical history and physical examination were taken from all patients with ESFT. A standard X-ray and magnetic resonance imaging (MRI) were performed to define the extent of the primary tumor. Tumor size was measured by MRI, defined as the longest diameter of the primary tumor. Plain chest X-ray and computed tomography (CT) of the chest were used to detect lung metastasis. A bone scintigraphy or gallium 67 scan for tumor survey was conducted to identify bone or soft tissue metastases. Baseline laboratory studies included complete blood cell and differential count, liver and renal function tests, and serum lactate dehydrogenase (LDH) level. Before the beginning of chemotherapy, patients were also examined for hepatitis B and C serology, as well as baseline 24-h urine creatinine clearance, hearing function, electrocardiogram, and echocardiogram. All patients were pathologically proven, which included immunohistochemistry, electron microscopy, and cytogenetic analysis, when available. An MRI and X-ray of the primary site, chest CT, and whole-body bone scan/gallium scan for tumor survey were assessed every 3 months during chemotherapy, 2 years after the end of chemotherapy, followed by twice a year for the next 3 years, and once a year for the remainder of the patient's life.

### Treatment

**FIGURE 1:**
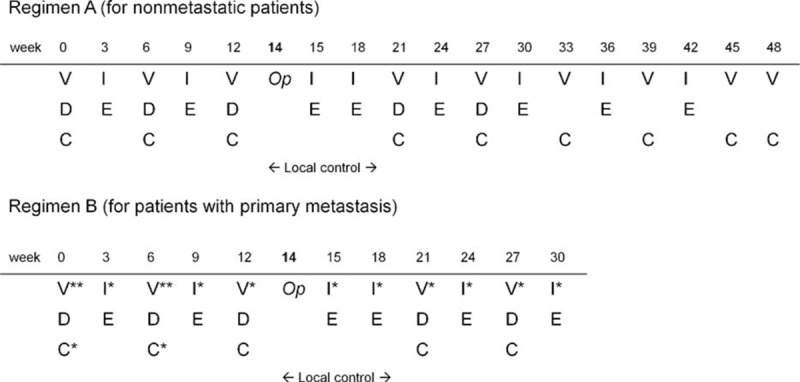
Chemotherapy schemes for patients with ESFT without metastasis (regimen A) or with metastasis (regimen B). *Op* operation, *V* vincristine (1.5 mg/m^2^, max. 2 mg, i.v. drip 15 min, once), *V*^∗∗^ vincristine (1.5 mg/m^2^, max. 2 mg, i.v. drip 15 min, weekly for 3 wk), *V*^∗^ vincristine (1.5 mg/m^2^, max. 2 mg, i.v. drip 15 min, once), *D* doxorubicin (37.5 mg/m^2^/d as a 48-h continuous i.v. infusion; total dose 75 mg/m^2^), *C* cyclophosphamide (1200 mg/m^2^ as a 1-h infusion), *C*^∗^ cyclophosphamide (2100 mg/m^2^/d as a 1-h infusion for 2 d; total dose 4200 mg/m^2^), *I* ifosfamide (1800 mg/m^2^/d as a 1-h infusion for 5 d; total dose 9000 mg/m^2^), *I*^∗^ ifosfamide (2400 mg/m^2^/d as a 1-h infusion for 5 d; total dose 12,000 mg/m^2^), *E* etoposide (100 mg/m^2^/d as a 1-h infusion for 5 d; total dose 500 mg/m^2^).

Patients who were diagnosed before 1997 received chemotherapy consisting of vincristine, doxorubicin, and cyclophosphamide (VDC). After 1997, the main chemotherapy regimens consisted of multiple cycles of VDC, alternating with Ifosfamide and Etoposide (IE) (Regimens A and B, for nonmetastatic and metastatic diseases, Figure 1). All protocols included preoperative and postoperative chemotherapy.

Informed consent for chemotherapy was obtained from every patient or patient's legal guardian prior to chemotherapy treatment. This study followed all the guidelines of TVGH. The chemotherapy protocols were reviewed and approved by the Cancer Treatment Quality Monitoring Board of TVGH.

After neoadjuvant chemotherapy, the surgical planning of primary tumor excision was determined by orthopedic surgeons, in accordance with the recommendations of a multidisciplinary tumor board. After a resection of the primary tumor, all metastases were surgically removed whenever possible. Radiotherapy would be performed postoperatively if the surgical margin was pathologically unclear or considered inadequate by the principle surgeon. The decision of whether to perform high-dose chemotherapy with autologous peripheral blood stem cell rescue (auto-PBSCT) varied according to the treatment period, the attending physician's experience/decision, the socioeconomic background of the family, and the payment provisions of Taiwan's National Health Insurance system. In most cases, auto-PBSCT could be 1 treatment option if the patient was considered to be at a high risk of relapse, but exhibited a positive response to neoadjuvant chemotherapy and underwent successful radical tumor excision.

### Statistics

The factors age, sex, primary tumor site, tumor size, tumor tissue of origin, LDH at diagnosis, surgical margins, and radiotherapy for local control (includes radiotherapy alone or radiotherapy postoperatively) were compared between the metastatic and nonmetastatic groups by performing the χ2 test or Fisher exact test. Overall survival (OS) was calculated as the time from the date of diagnosis to that of death or last patient contact. Progression-free survival (PFS) was taken to be the time from diagnosis until disease progression, relapse, the development of a second malignant neoplasm, death, or last patient contact. The OS and PFS curves were estimated according to the Kaplan–Meier method and compared using the log-rank test. The parameters evaluated in univariate analysis were used as explanatory variables in the Cox regression models of OS and PFS and the final multivariate models. A 2-sided *P* value of <0.05 was defined as statistically significant. SPSS (version 19) software was used for data analyses. The data of patients used in these analyses were last updated on September 17, 2014.

## RESULTS

### Patient Characteristics

**FIGURE 2:**
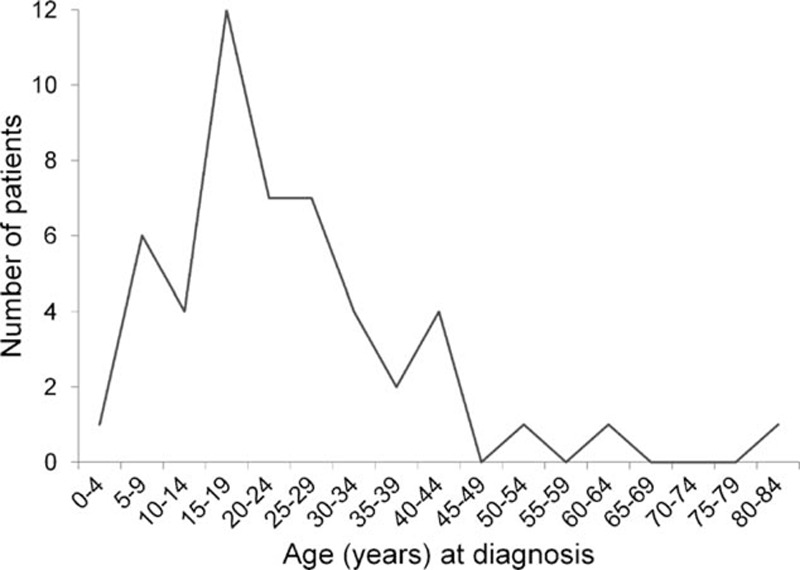
Number of patients with ESFT according to age.

Fifty-seven newly diagnosed patients with primary ESFT who were admitted to TVGH between February 1991 and March 2014 were retrospectively evaluated for eligibility. Seven patients were excluded because of loss to follow-up (n = 6) or transfer to another hospital (n = 1). Fifty patients were recruited, and their characteristics are listed in Table 1. The median age at diagnosis was 20.2 years (range, 3.4–80.5 y), with a male-to-female ratio of 1:1. Patient numbers according to age are depicted in Figure 2. The primary tumor sites included axial bones (n = 28, 56%), extremities (n = 15, 30%), and soft tissue (n = 7, 14%). The median follow-up time was 1.8 years (range, 0.4–9.9 y). Regarding the patients, 21 of the 50 (42%) were metastatic at diagnosis. Of the 21 patients with primary metastasis, 10 had bone metastasis (47.6%), 9 had pulmonary metastasis (42.8%), 1 had both bone and lung metastases (4.8%), and 1 had brain metastasis (4.8%). The mean of LDH in 31 assessable patients was 293.3 U/L (range, 117–759 U/L). Patients with metastasis have significantly higher levels of LDH compared with those without (363.4 vs 249.1 U/L, *P* = 0.031, n = 12 and 19, respectively, independent sample *t* test). For the local control, 38 of the 50 patients (76%) underwent tumor excision surgery, and 17 received additional radiotherapy postoperatively. Eight patients (16%) received radiotherapy only. Neither surgery nor radiotherapy was performed for 4 patients (8%). Regarding the systemic chemotherapy for the patients, 49 of 50 were diagnosed after 1997, and received chemotherapy regimens with VDC-IE (Figure 1). Only 1 patient was diagnosed before 1997, and this patient received chemotherapy with VDC only. After the first 2 to 3 courses of chemotherapy, 11 of the 50 patients (22%) had also received auto-PBSC harvest, and subsequently received high-dose chemotherapy with auto-PBSCT at the end of all courses of chemotherapy.

### OS and PFS

**FIGURE 3:**
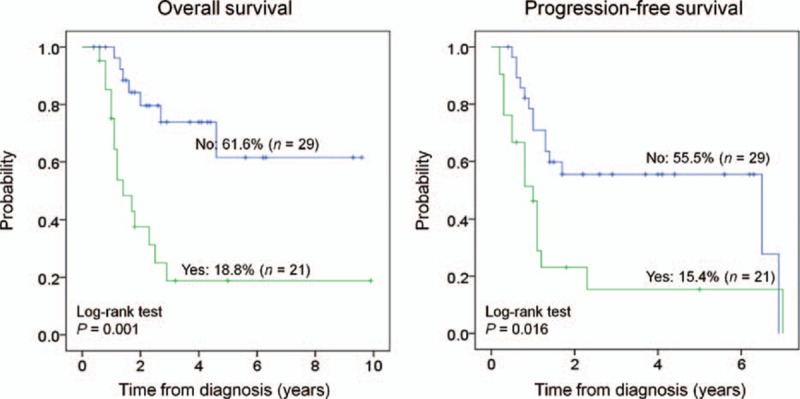
Kaplan–Meier curves comparing progression-free survival and overall survival in patients with ESFT with (yes) or without (no) metastasis.

The 5-year OS and PFS were 43.2% and 39.4% for patients, respectively. Twenty-two patients had died by the final follow-up; all causes of death were related to disease relapse or progression. Thirty-one patients (62%) experienced disease relapse or progression. The median time to progression was 0.9 years (range, 0.2–7 y). Regarding the patients, 9 of 31 (29%) with cancer progression were still alive at the time of the study. However, only 2 of the 17 patients with primary metastasis whose disease progressed survived (11.8%). The estimated 5-year OS and PFS were 61.6% and 55.5% for patients who were nonmetastatic, and 18.8% and 15.4% for patients who were metastatic, respectively (Table 2, Figure 3).

**TABLE 2:**
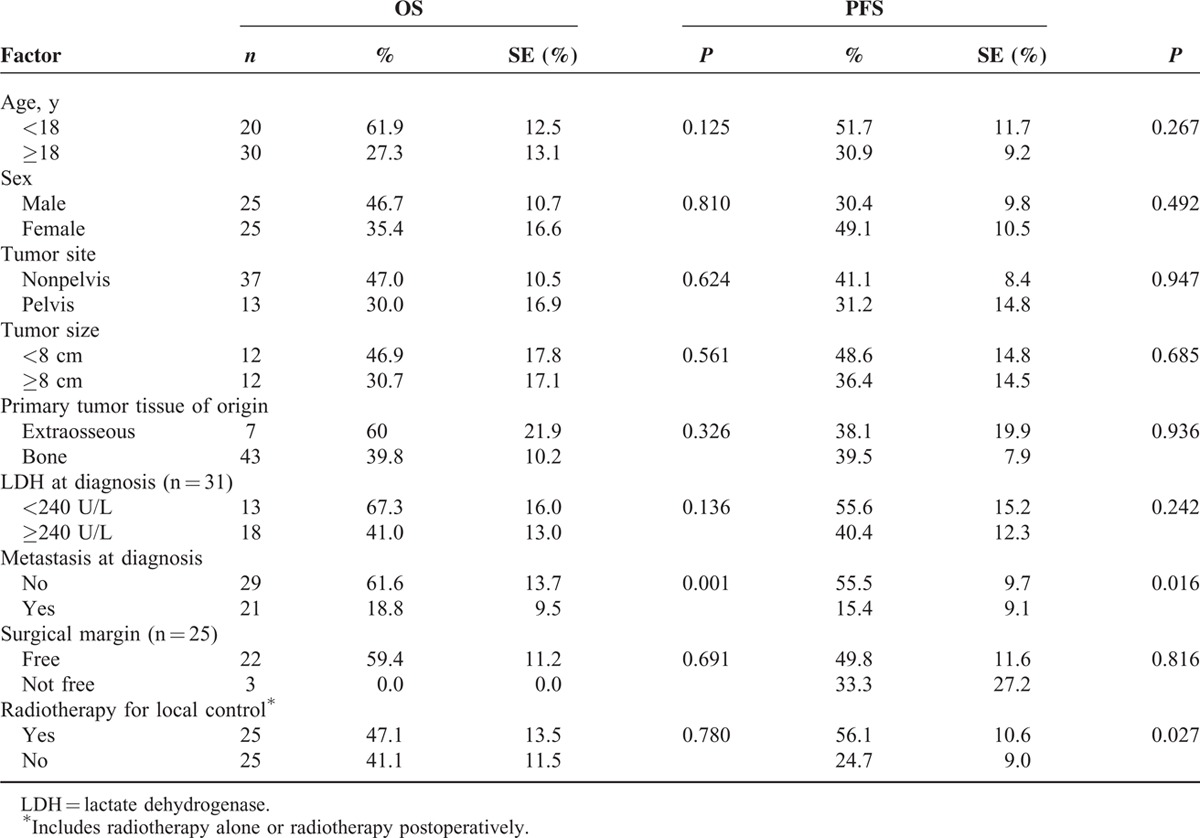
Univariate Analysis of Prognostic Factors for 5-Y OS and PFS Based on Kaplan–Meier Estimates and Log-Rank Tests for Patients With ESFT (n = 50)

In univariate analysis, only 2 factors were significantly correlated with survival (Table 2). Metastasis at diagnosis was adversely correlated with OS (*P* = 0.001) and PFS (*P* = 0.016). Radiotherapy in the local control was significantly associated with an improved PFS (*P* = 0.027). In multivariate analysis using the Cox regression model (Table 3), primary metastasis was significantly associated with OS and PFS (hazard ratio (HR) 9.66 and 3.73, *P* <0.001 and *P* = 0.004, respectively). Radiotherapy in the local control was significantly associated with PFS (HR 2.58, *P* = 0.028). Moreover, primary tumor site that was over the pelvis exhibited a borderline association with OS (HR 3.13, *P* = 0.056).

**TABLE 3:**
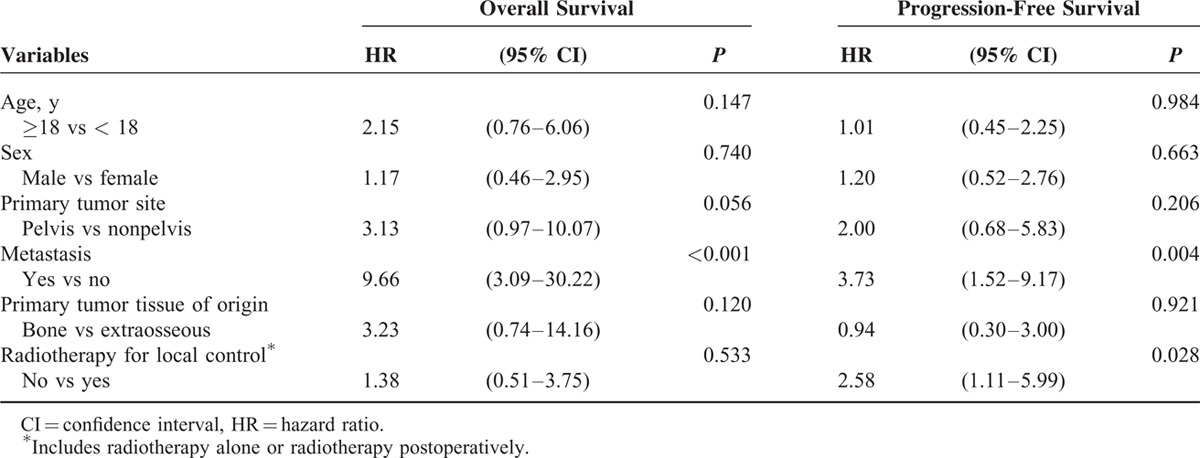
Multivariate Analysis of Variables and Survival in Patients With ESFT (n = 50) According to Cox Regression Models

## DISCUSSION

This study presented the first long-term outcome of ESFT at our institution. Although we compared the 5-year OS for nonmetastatic ES among countries, the result (61.6%) in this study was similar to a multi-institutional analysis of ES of bone in Japan (Japanese Musculoskeletal Oncology Group, 5-y OS 54.9%; event-free survival 67.6%, and 41.2% for patients who received vincristine, actinomycin-D, cyclophosphamide, and doxorubicin (VACD) plus IE or VACD chemotherapy alone),^8^ but significantly poorer than most of the results obtained from contemporaneous Western studies, where the 5-year OS ranged from 65% to 83%.^9–11^ Moreover, a report from the Children's Oncology Group demonstrated a 5-year OS for patients <50-year old, with localized extradural ES of 77% to 83%.^9^ Results from St. Jude Children's Research Hospital were even more favorable, showing a 5-year OS of 88.1% for patients <14-year old with localized nonpelvic tumors.^10^ Surgical principles and techniques are more unified and standardized in a single institutional study (like ours) than in multi-institutional series. Our past findings revealed a 5-year OS and PFS of 77% and 70%, respectively, and a limb salvage rate of 97.3% for pediatric osteosarcoma of the extremities;^12^ this was comparable to the most recent results from EURAMOS-1.^13^ The surgical team for ESFT in the current study was the same as in the previous report, which was mainly conducted by experienced orthopedic surgeons devoted to musculoskeletal tumors.^14,15^ Furthermore, the majority of our patients received chemotherapy regimens consisting of VDC plus IE;^16^ this 5-drug regimen has been demonstrated to be superior to VDC alone, and has been widely used in Western countries.^9,17,18^ While controlling for surgical technique and chemotherapy regimens, the poorer treatment outcome in our series might be attributable to other factors, including genetics. Ethnicity has been reported to be 1 risk factor in developing ESFT; Caucasians have the highest incidence of ES, followed by Asians/Pacific Islanders and African Americans, reported by a study using data from the Surveillance, Epidemiology, and End Results database.^3^ Another study from the Surveillance, Epidemiology, and End Results analyzed 1715 patients of ES and reported that OS was significantly poorer for black, Asian, and white Hispanic patients compared with white non-Hispanic patients (*P* = 0.0031, *P* = 0.0182, and *P* = 0.0051, respectively).^4^ In Taiwan, up to 98% of the population is Han Chinese. One previous population-based analysis disclosed that the ES incidence rate in Taiwan was 2- to 3-fold lower than the United States and England.^5^ In China, poorer outcome of ESFT with 5-year OS ranged from 28.6% to 33.3% for nonmetastatic patients,^19,20^ and rarity of ESFT have also been reported.^21^ The results from the current study, along with those previous reports, are critical evidence of racial disparity in ESFT incidence and treatment response between Han Chinese and Caucasian people, and suggest that genetic factors play a role in both the pathogenesis of ESFT and chemotherapy susceptibility.

The presence of metastases at diagnosis is the key adverse prognostic factor for ESFT.^8,10,16,18,22–26^ Approximately 25% of patients with ESFT were metastatic at diagnosis, as reported by a population-based study from the United States.^11^ A large study from Japan determined that 16.9% (41 of 243 patients) of patients with ESFT were metastatic at diagnosis.^8^ Compared with these reports, the current findings revealed an extraordinarily high rate of metastatic disease at diagnosis: 42% (21 of 50 patients). The 5-year OS and PFS for metastatic ESFT were 18.8% and 15.4%, respectively, over 40% lower than nonmetastatic patients (*P* = 0.001 and *P* = 0.016, respectively). In the 21 patients with primary metastasis, 47.6% had bone metastasis, 42.8% had pulmonary metastasis, 4.8% had both bone and lung metastasis, and 4.8% had brain metastasis. The prognosis of ESFT with bone metastasis was even worse than for lung metastasis.^18^ In this study, the rate of bone metastasis (52.4%) exceeded the rate of lung metastasis (47.6%), which indicated more aggressive disease behavior, poorer outcome, and the need for more intensive treatment. The reasons for the high rate of primary metastases demonstrated in patients with ESFT require further investigation. Possible reasons include bias associated with patients’ hospital selection or different characteristics of the disease in Taiwanese patients with ESFT. Further investigation of tumor genetics and new therapeutic strategies from biological studies, and development of more effective systemic chemotherapy, especially for metastatic ESFT in Taiwan, is required.

ESFT is considered a radiosensitive tumor. Previous studies have indicated that radiotherapy had an imperative role in local disease control, especially in patients who could not undergo radical surgery or who had received surgical excision with inadequate surgical margins.^26–29^ The results from the CESS 86 trial for ES of bone demonstrated radiation therapy yielded relapse-free and OS rates comparable to radical surgery under the given selection criteria for local therapy.^30^ In this study, the 5-year PFS in patients who received radiotherapy in local treatment (including radiotherapy alone or radiotherapy postoperatively) was significantly better than those who did not (56.1 vs 24.7%, *P* = 0.027). Consistent with previous reports, the results from this study suggested that radiotherapy could improve PFS and could be considered first line treatment. In Taiwan, a nationwide cohort study revealed that 18.3% of children received traditional Chinese medicine, especially when they had a musculoskeletal illness.^31^ Approximately 30% of our pediatric osteosarcoma patients received prior manipulative therapy,^12^ and it has been proven to be an independent risk factor when performed before osteosarcoma diagnosis, as reported elsewhere.^32,33^ Further studies are required to investigate the impact of traditional Chinese medicine and prior manipulative therapy on ESFT in Taiwan to determine if more aggressive radiotherapy should be performed in this group of patients to prevent local recurrence and subsequent systemic metastasis.

Previous studies, which have used various cutoff values for age, have suggested adverse effects of older age on outcome.^16–18,23,26,28^ Consistently, our study has measured that the OS and PFS rates were 20% to 30% lower in patients aged >18 years than in children, but these results were statistically nonsignificant (Table 2). In addition, older patients in this study (median age 20.2 y) might be 1 factor contributing to a poorer outcome compared with other studies (median age 13.7–15 y).^10,34^ Tumor location and size were also associated with the prognosis.^8,16–18,23,34^ Several studies have determined that pelvic ESFT resulted in a worse outcome because of difficulties in the surgical control of the primary tumor, advancement of the disease, and the distinct biology of the pelvic tumor.^8,16–18,23,34^ Studies have indicated that tumors larger than 8 cm are significantly associated with poor survival,^16,23^ whereas others have indicated no survival difference.^22,27,28^ The results of the current analysis revealed that the survival rate was 10% to 17% lower in pelvic tumors compared with nonpelvic tumors, and 12% to 16% lower with a tumor size >8 cm compared with a tumor size <8 cm; however, neither of these results were statistically significant (Table 2).

This study has several limitations. Bias resulted from the pure retrospective design, and the study period was long, and included improvements in diagnostic technology, surgical skills, radiotherapy, supportive care, and teamwork operation over time. Moreover, Taiwan's National Health Insurance program, which was implemented in 1995, and promises equal access to health care for all citizens, has been known to have a great impact on patient behavior and the Taiwanese medical system in numerous aspects compared with before 1995. In addition, the limited case number associated with the rarity of ESFT might reduce the statistical power, especially when grouping patients into subgroups to conduct comparisons by prognostic factors.

In conclusion, this is the first study in Taiwan to evaluate the prognostic factors and long-term survival of patients with ESFT. Consistent with previous reports, the findings indicated that primary metastases were the key factor adversely associated with OS and PFS. The high rate of primary metastases and poorer outcomes of nonmetastatic ESFT compared with the outcomes of patients from Western studies using similar VDC-IE regimens, along with previously reported low rates of ESFT in Taiwanese people, suggest that genetic factors play a role in the pathogenesis of ESFT and chemotherapy pharmacokinetics and pharmacodynamics. Radiotherapy was associated with improved PFS, and might be considered in local treatment more aggressively in Taiwanese patients with ESFT, especially when these patients have received prior manipulation before the diagnosis of ESFT. Further randomized-controlled trials to clarify the impact of dosages and cycles of IE, in addition to VDC, on systemic control of ESFT (e.g., less cycles of VDC, and more cycles of IE) are warranted, especially for the Asian population.
